# Retrospective Study of Clinical and Genetic Profiles of Alpha‐Mannosidosis Patients From the UAE

**DOI:** 10.1002/jmd2.70001

**Published:** 2025-02-06

**Authors:** Ali K. Saad, Tasneem Al‐Hammadi, Shaikha Al‐Ameri, Aisha Al‐Shamsi, Noura Al‐Dhaheri, Amal Al Tenaiji, Fatma Al Jasmi

**Affiliations:** ^1^ Department of Genetics and Genomics College of Medicine and Health Sciences Al Ain UAE; ^2^ Genetic Division, Department of Pediatrics Tawam Hospital Al Ain UAE; ^3^ Department of Pediatrics SKMC Abu Dhabi UAE

**Keywords:** alpha‐mannosidosis, developmental delay, hearing loss, lysosomal storage disease, *MAN2B1*

## Abstract

Alpha‐mannosidosis (AM; OMIM 248500) is a rare autosomal recessive lysosomal storage disorder caused by mutations in *MAN2B1*, which codes for the lysosomal alpha‐mannosidase enzyme (LAMAN; EC:3.2.1.24). Clinical characteristics include developmental delay, hearing impairment, and recurrent infections. A retrospective analysis of nine case series of patients with AM (23 months–42 years) from six consanguineous families in the United Arab Emirates (UAE) was conducted. In two Emirati families, homozygous nonsense mutations were present: c.2368C> T, p.(Gln790*) and c.2119C> T, p.(Gln707*). Further, in the Emirati and Syrian families two splicing variants c.2356‐2A>G and c.1929‐2A>G were present, respectively. All patients had infantile‐onset and common clinical features, including coarse facies, developmental delays, hearing loss, and recurrent infections. Macrocephaly was observed in all patients with documented head circumference, except one microcephalic patient who had a dual genetic diagnosis. Hepatosplenomegaly and autoimmune diseases were reported in one and three patients, respectively. Additionally, psychiatric manifestations were noted in two adult patients. The mean age of diagnosis was 14 years for adults (> 16 years) and 2 years for pediatric patients (< 16 years). Significant diagnostic delay comparing older and younger generations is likely due to the increasing awareness of genetic disorders and the availability of genetic testing. In terms of treatment, enzyme replacement therapy (ERT) was administered to two patients, alleviating recurrent infections. Two patients underwent hematopoietic stem cell transplantation (HSCT), whereas one patient underwent combined ERT and HSCT. This retrospective analysis identified different truncating mutations associated with early‐onset AM. The clinical presentations of these mutations range from attenuated to moderate. Our analysis clearly highlights the high birth prevalence of AM in the UAE, indicating the need for awareness and genetic counseling for prevention.

**Summary**

- The study provides a detailed description of nine cases from six different families presenting with a rare lysosomal storage disorder, namely, alpha‐mannosidosis.
- All patients had an infantile‐onset disease with severe homozgous mutations.
- Most of patients presented with developmental delay, hearing loss, recurrent infection, coarse facies as well as macrocephaly.

**Abbreviations**
ABR: auditory brainstem response
AM: alpha‐mannosidosis
BMT: bone marrow transplantation
ERT: enzyme replacement therapy
ES: exome sequencing
GVHD: graft‐versus‐host disease
HSCT: hematopoietic stem cell transplantation
NGS: next‐generation sequencing
UAE: United Arab Emirates

## 1 Background

Alpha‐mannosidosis (AM; OMIM 248500) is a rare autosomal recessive lysosomal storage disorder that affects 1 in 0.5–1 million individuals worldwide [1, 2, 3, 4, 5]. A high prevalence of autosomal recessive disorders in the United Arab Emirates (UAE) coincides with high rates of consanguineous marriages in the country [5, 6]. Reportedly, the prevalence of AM in the UAE is four times higher than global records [7].

Biallelic pathogenic variations in *MAN2B1* cause AM [2, 8, 9]. This gene codes for a lysosomal alpha‐mannosidase enzyme—EC 3.2.1.24 [2, 9]. *MAN2B1* is involved in the lysosomal degradation of mannose‐rich oligosaccharides during glycoprotein catabolism [2, 10, 11]. Malfunction or deficiency of the α‐mannosidase enzyme results in the accumulation of its substrates, causing lysosomal dysfunction and multisystemic manifestations [1, 12]. Analysis of accumulating oligosaccharides in urine and plasma is used as screening tools and initial baseline assessment for diagnosed patients [13, 14, 15]. Levels of serum/urine oligosaccharides can also be used for monitoring treatment effectiveness in enzyme replacement therapy (ERT), as recommended by a recent Delphi consensus study [14]. Residual enzyme activity in leukocytes is the gold standard for diagnosing AM [1]. Moreover, genetic testing is performed to identify causative mutations and confirm the diagnosis of AM in line with recent recommendations [2, 14]. Advances in next‐generation sequencing (NGS) have facilitated the diagnosis of rare diseases such as AM by directly opting for genetic testing in suspected cases.

AM has a heterogeneous clinical presentation with varying severities and symptom onset, complicating early diagnosis [16]. The clinical presentation of AM resembles mucopolysaccharidosis (MPS), which could further delay diagnosis [17]. Global developmental delay is the primary presentation of AM, and some patients may present with ataxia and/or psychosis [4, 18, 19]. Musculoskeletal problems—such as arthritis, myopathy, and scoliosis—further restrict patient mobility [4, 20]. With disease progression, some patients may become wheelchair‐bound [16]. Hearing loss is another distinct clinical feature of AM [4, 8, 12, 19], typically due to sensorineural damage and/or secondary to recurrent otitis media, which causes conductive dysfunction [1, 12]. Speech delay is typically associated with hearing loss [12, 19]. Recurrent infections and high vulnerability to autoimmune diseases such as Hashimoto's thyroiditis and systemic lupus erythematosus have been reported [4]. Comprehensive evaluation of newly diagnosed patients is highly recommended for affected organ systems involved in AM, including audiological, neurological, musculoskeletal, cognitive, psychiatric, immunological, cardiac, and respiratory systems. Organ system evaluations may differ in priority between pediatric and adult patients [14]. Depending on the disease course or patient age, further referral to specialists may be required (neurologist, orthopedist, psychologist, physiotherapist, and immunologist) [14].

**FIGURE 1:**
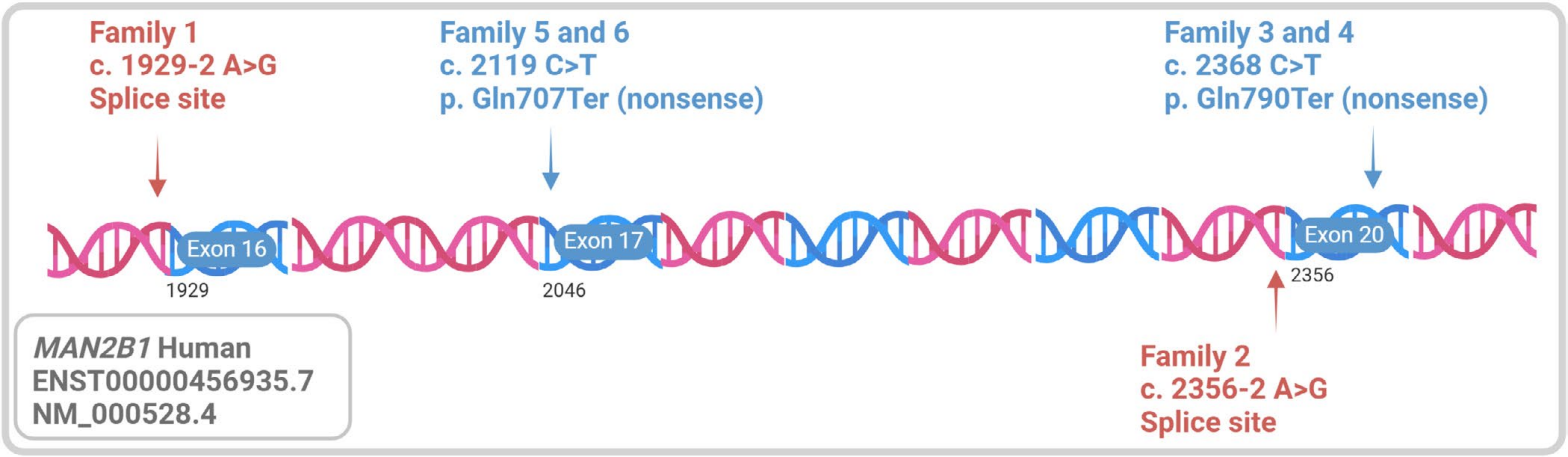
Schematic description of the DNA stretch of *MAN2B1* with the location and type of the variant for each family; transcript information and exon numbers were calculated from Ensembl using the clinically relevant transcript. Created in BioRender. https://BioRender.com/v81n231.

ERT can aid in treating AM by replenishing the body with alpha‐mannosidase [21, 22]. ERT administration for at least 2 years has been shown to be effective in ameliorating immunodeficiency and hearing loss in concordance with a reduction of serum oligosaccharide levels [19, 23]. Moreover, a recent long‐term follow‐up study (spanning up to 12 years) on patients treated with Velmanase alfa demonstrated a delay in the progression of non‐central nervous system disease across all age groups [23]. Many pediatric patients experienced improvements in motor (enhanced performance in the 6‐min walk test [6MWT] and 3MSCT) and pulmonary functions (forced vital capacity [FVC], % predicted), while adults showed stabilization in these functional outcomes during prolonged treatment [23]. Additional non‐age–related benefits include sustained clearance of serum oligosaccharides and elevated serum IgG levels. Unfortunately, effectiveness of ERT is limited owing to its inability to cross the blood–brain barrier (BBB) [21, 22, 24]. Thus, bone marrow (BMT) and hematopoietic stem cell (HSCT) transplantations are the primary options for several lysosomal storage disorders, including severe AM [22, 25]. That said, BMT/HSCT is associated with complications, namely: (1) graft‐versus‐host disease (GVHD) that may have lethal consequences; (2) rejection of the transplant; and (3) prophylactic immunosuppressant usage, which elicits substantial side effects [1, 22, 26]. The first‐line intervention in preventing graft rejection and GVHD aims to improve histocompatibility or reduce immune reaction by matching all 4–5 HLA genes between donor and recipient (8/8 allele match in USA standards or 10/10 allele match in European standards) [27]. Monitoring engraftment and related GVHD posttransplantation by a specialist is agreed upon in the Delphi consensus study [14]. Furthermore, BMT/HSCT should be administered early to preserve neurological function [16]. The combination of therapies (ERT and HSCT) is a promising way to treat neurological and non‐neurological symptoms [28]. The rarity of the disease, complicated differential diagnosis, and variable onset of symptoms may delay diagnosis and early treatment [12]. At present, there is no clear genotype–phenotype correlation that enables early diagnosis of the disease [2, 8]. Herein, we retrospectively report nine cases of confirmed AM in the UAE to further document the genetic variations (Figure 1), clinical presentations (Table 1), onset of symptoms in AM, and their treatment.

**TABLE 1:** Clinical and genetic summary of nine cases with AM.

Homozygous NM_000528.3 (MAN2B1)	c.1929‐2A>G	c.2356‐2A>G		c.2368C> T (p.Gln790^a^)			c.2119C> T (p.Gln707^a^)		
Mutation type	Splicing	Splicing		Nonsense			Nonsense		
HGMD	DM	DM?		DM			DM		
ClinVar	Pathogenic	Pathogenic		Pathogenic			Pathogenic		
Family	**Family 1**	**Family 2**		**Family 3**	**Family 4**		**Family 5**	**Family 6**	
Parental consanguinity	Yes	Yes		Yes	Yes		Yes	Yes	
Nationality	Syrian	United Arab Emirates		United Arab Emirates	United Arab Emirates		United Arab Emirates	United Arab Emirates	
Patient	**1.1**	**2.1**	**2.2**	**3.1**	**4.1**	**4.2**	**5.1**	**6.1**	**6.2**
Age (2024)	43 years	10 years	18 years	4 years	27 years	23 years	10 years	21 years	15 years
Gender	Male	Male	Female	Male	Female	Female	Male	Male	Male
Height at current age	160.5 cm	123 cm	155 cm	117 cm	145 cm	151.5 cm	144 cm	164 cm	153 cm
Weight at current age	62 kg	28.5 kg	78 kg	23 kg	53 kg	52.6 kg	36 kg	54.2 kg	37.7 kg
Age of onset	At birth	At birth	2 years	At birth	6 months	At birth	At birth	6 months	At birth
Age at diagnosis	37 years	3 years	10 years	20 months	13 years	10 years	5 years	NA	At birth
Speech and language	Few words	Vocalizing only	Special education	One word	Special education	Few words	Speech therapy	Clear speech with immature language structure	Doing well in mainstream school
IQ	NA	NA	58–77	NA	40–55	40–55	77	NA	NA
Psychiatry symptoms	Yes	No	No	No	No	Yes	No	NA	NA
Hearing loss	Yes	Yes	Yes	Yes	Yes	Yes	Yes	Yes	NA
Recurrent otitis media	Yes	Yes	Yes	Yes	Yes	No	Yes	Yes	NA
Respiratory tract infections	No	Yes	Yes	Yes	Yes	Yes	Yes	Yes	NA
Autoimmune	No	No	Hashimoto thyroditis	No	Hashimoto thyroditis	Hashimoto thyroditis	No	No	Hashimoto thyroditis/Graves' disease
Hepatosplenomegaly	Yes	Yes	No	Yes	No	No	No	NA	NA
Hypotonia	Yes	Yes	No	No	No	No	No	No	No
Ataxia	Yes, worsening	No	No	No	No	Yes	No	No	No
Ambulation	Walking with support (regression)	Wheelchair	Walking and running	Walking with support	Walking	Walking with support	Walking and running	Walking	Walking
Feeding difficulties	Yes	Yes	No	No	No	No	No	No	No
Height at current age	160.5 cm	123 cm	155 cm	117 cm	145 cm	151.5 cm	144 cm	164 cm	153 cm
Weight at current age	62 kg	28.5 kg	78 kg	23 kg	53 kg	52.6 kg	36 kg	54.2 kg	37.7 kg
Head size	Macrocephaly	Microcephaly^a^	NA	Macrocephaly	NA	NA	Macrocephaly	NA	Normocephalic
Coarse facial features	Yes	Yes	No	Yes	Yes	Yes	Yes	NA	Yes
Scoliosis	No	Yes	Yes	No	Yes	Yes	No	NA	Yes
Cardiac anomaly	No	Tetralogy of Fallot (another genetic disease)^a^	MVR corrected	No	No	Trace MVR	Trivial MVR	No	MVR
Management	Supportive	ERT at 7 years	ERT at 15 year	ERT+ 10/10 HSCT at 3 years	Supportive	Supportive	Supportive	9/10 BMT at 3 years	5/6 UCB transplant at 1 year
Posttreatment follow‐up	NA	No recurrent infections	No recurrent infections	Improved hearing, low IgG	NA	NA	NA	Improved sociability and speech/ no regression	6‐MWT performance is 52% of norm, mainstream school, and pleuroparenchymal fibroelastosis

Abbreviations: BMT, bone marrow transplant; DM, disease‐causing mutation; DM? = likely disease causing; ERT, enzyme replacement therapy; HSCT, hematopoietic stem cell therapy; NA, not available; UCB, umbilical cord blood.

^a^ This patient had a dual genetic diagnosis, microcephaly, and cardiac condition as a result of *TMEM260*.

## 2 Methods

### 2.1 Ethical Approval

This study was approved by the Abu Dhabi Health Research and Technology Committee, reference number DOH/CVDC/2023/834. Affected patients were identified by the metabolic teams at Tawam Hospital and Sheikh Khalifa Medical City (SKMC), Abu Dhabi, UAE, for clinical evaluation and follow‐up. Tawam Hospital and SKMC are the referral hospitals for genetic diseases in the UAE.

### 2.2 Data Source and Limitations

Patient files were accessed retrospectively through Salamtak—an electronic medical record platform. The inclusion specification was the genetic confirmation of AM through genetic testing (Sanger or exome sequencing). This study is limited owing to its retrospective nature; thus, patient information outside the Salamtak platform was excluded.

### 2.3 Live Birth Prevalence of AM in UAE

There are various ways of calculating prevalence. The live birth method for calculating prevalence was used in earlier studies on AM [7, 29, 30, 31]. The number of patients born in UAE (all patients except 1.1 who were born abroad) is divided by the number at risk in the population. At risk is calculated by the live births in UAE in the period between the date of birth of the oldest patient (1997) and the time of birth of the youngest (2020). The result is multiplied by 100 000 to find the prevalence per 100 000. All the patients born in this cohort are Emiratis, so the total live births of Emirati only are used (i.e., 700 303 live births 1997–2020) rather than total live births in the entire country. The source of live birth numbers is from the Federal Competitiveness and Statistics center (FCSC) website (uaestat.fcsc.gov.ae/en).

## 3 Results

This section is devoted to the retrospective reporting of all relevant information from each patient file, including their genetic variants (Figure 1). Table 1 summarizes the clinical and genetic characteristics of each patient. All patients in this retrospective cohort came from consanguineous families.

### 3.1 Family 1

#### 3.1.1 Case 1.1

This is the case of a 42‐year‐old Syrian male whose family history includes two healthy siblings. Additionally, the patient had a history of craniosynostosis, requiring surgical intervention at 1 year of age. He also experienced sensorineural and conductive hearing loss at 7 months of age, which was managed with hearing aids. Recurrent ear infections had persisted since his childhood. The patient had a history of tonsillectomy and adenoidectomy, as well as umbilical hernia and inguinal hernia surgery. At 20 years of age, he developed spinocerebellar ataxia, which progressively worsened over time. During adulthood, cerebral and cerebellar atrophy, along with white matter changes, were observed. His motor function began to decline at 39, leading to complete dependence on his family for mobility, and he became wheelchair‐bound by 40 years of age. Additionally, he experienced feeding difficulties, including occasionally choking on food. The patient also had a history of multiple fractures, with a DEXA scan confirming osteopenia. Since his third decade, psychiatric symptoms had progressively worsened, including severe depression, self‐harm, and mood swings.

*Genetic testing*: At the first year of life, he was suspected of having MPS, and enzyme testing was negative. At the age of 37 years, exome sequencing (ES) revealed a homozygous mutation c.1929‐2A>G in *MAN2B1*, affecting the splice acceptor site at Exon 16 and confirming the diagnosis of AM. *Treatment and follow‐up*: ERT was not implemented because of limited medical insurance coverage.

### 3.2 Family 2: Siblings (Cases 2.1 and 2.2)

#### 3.2.1 Case 2.1

This case is of a 9‐year‐old male. He had an older sibling with the same clinical presentation (Section 3.2.2 below). Another sibling passed away at the age of 4 months due to cyanotic congenital heart disease. The family had two other healthy children. Born with severe respiratory distress and hypoxia, he was antenatally diagnosed with pentalogy of Cantrell, tetralogy of Fallot, and omphalocele. He underwent open‐heart surgery and surgical repairs for the omphalocele and a diaphragmatic hernia at the age of 8 months. He exhibited feeding difficulties and failure to thrive, which was reported at the age of 2 months. He was further diagnosed with congenital laryngomalacia. He experienced recurrent ear and chest infections, including bilateral otitis media with effusion, leading to bilateral hearing impairment that required hearing aids. His speech development was minimal, limited to nonverbal vocalizations. Esotropia was noted in both eyes, and a brain CT scan revealed agenesis of the corpus callosum. Additionally, he had hypotonia, motor impairments, and was wheelchair‐bound. Bone abnormalities, including osteoporosis and rickets, were identified, for which appropriate supplements were administered. Cryptorchidism was also reported.

*Genetic testing*: At 3 years of age, genome sequencing revealed a homozygous, pathogenic, and splice‐altering variant c.2356‐2A>G in *MAN2B1*, confirming the diagnosis with AM. Additionally, a homozygous frame‐shifting deletion mutation c.1688del p.(Thr563Lysfs*32) was noted in the *TMEM260* gene, which was linked to the severe cardiovascular presentation. Other genetic mutations include CNV at 11p15.3‐p15.2 of uncertain significance. This case was published earlier by Bertoli‐Avella et al. without a full description of patient history, therapy, follow‐up, and his sibling's case [32]. *Therapy and follow‐up*: Velmanase alfa was initiated at the age of 7 years, reducing recurrent infections and preventing associated hospitalizations. Other interventions included physiotherapy twice a week and occupational and speech therapy once per week.

#### 3.2.2 Case 2.2

An 18‐year‐old female and a sibling of Section 3.2.1. The patient's medical records revealed an uneventful antenatal and perinatal history. Parents had noted hearing problems since the first year of life, but the patient did not receive appropriate medical attention until 8 years of age, when she was diagnosed with sensorineural hearing loss and subsequently started using hearing aids. She had recurrent chest infections in the initial years of life; she was diagnosed with asthma and was on prophylaxis until she was 3 years old. Furthermore, she underwent tonsillectomy at 2 years of age because of recurrent tonsillitis. Astigmatism was noted in both eyes. She had learning disabilities, delayed speech, and an IQ of 58–77, requiring special education to finish school, but she was socially active. She was diagnosed with a congenital mitral valve anomaly and mitral valve regurgitation and underwent surgery when she was 2 years old; subsequent echocardiograms confirmed stability. At 8 years of age, she was diagnosed with Hashimoto's thyroiditis and was managed with levothyroxine. Immunological assessment showed IgA and IgG deficiencies and neutropenia. Orthopedic intervention was prescribed for scoliosis with thoracolumbar curvature.

*Genetic testing*: Her case was diagnosed around 10 years ago when she underwent family screening, which reported a homozygous mutation c.2356‐2A>G in *MAN2B1*. She was heterozygous for the TMEM260 c.1688del p.(Thr563Lysfs*32) variant. *Treatment and follow‐up*: ERT (Velmanase alfa) was initiated at 15 years of age, which resulted in weight gain, better energy, fewer episodes of recurrent infection, and improvement in immunoglobulin and neutrophil count.

### 3.3 Family 3

#### 3.3.1 Case 3.1

**FIGURE 2:**
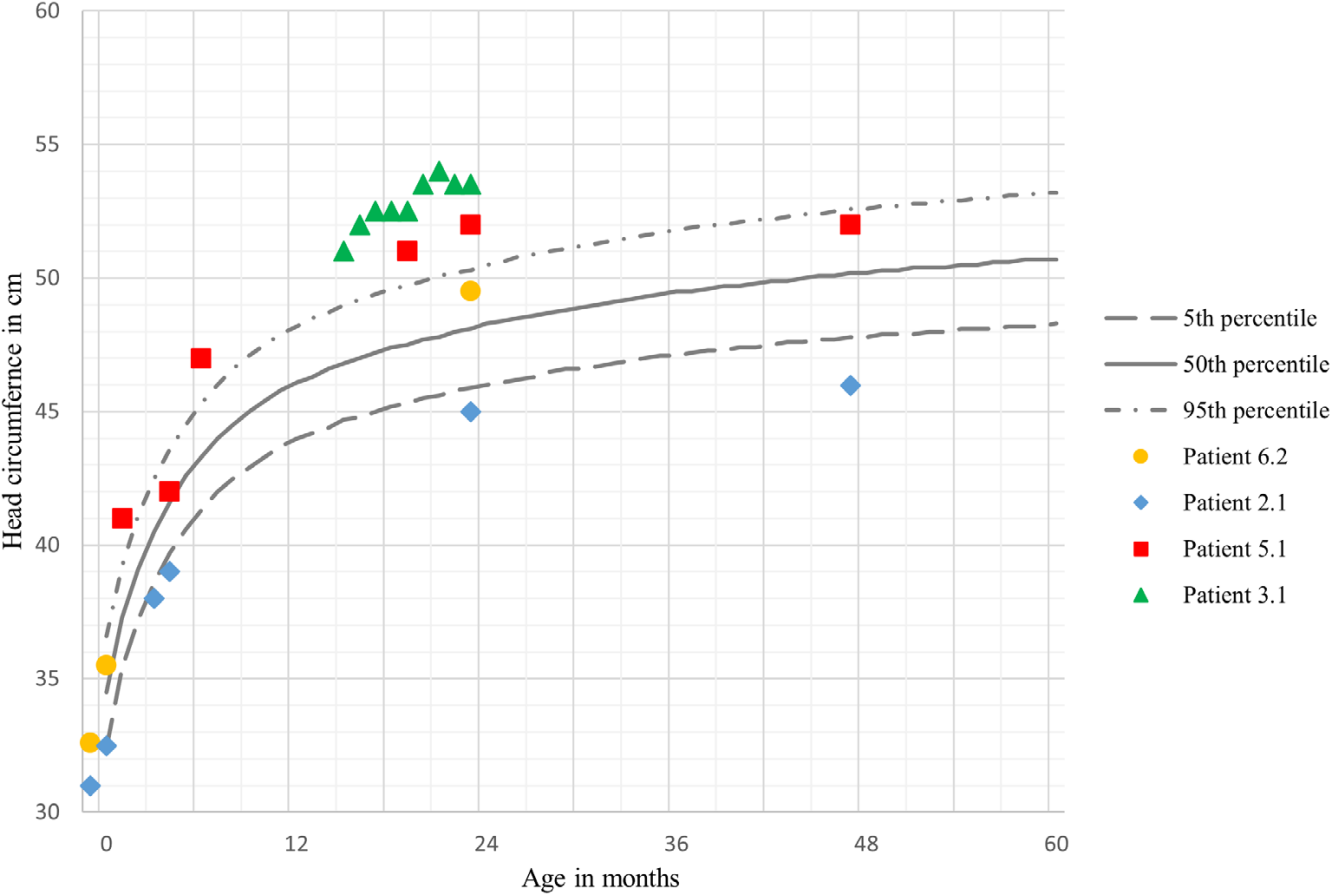
Head circumference change in centimeters with age in months up to 60 months for male patients based on WHO charts; lines represent 5th, 50th, and 95th percentiles as depicted in the legend on the right. Each patient is represented by a different shape used to plot readings with age.

A 4‐year‐old male with a negative family history of developmental delay, speech delay, or hearing loss. Recurrent infections and subtle dysmorphic features had been observed since the age of 6 months. He had macrocephaly at 16 months—which was 53.5 cm in size—and his head circumference, weight, and height were above 99th percentile (Figure 2). The developmental history indicated gross motor, fine motor, and speech delays. At 18 months, his family observed hearing impairment. Moreover, the patient visited an otolaryngologist multiple times for hearing impairment and adenoid hypertrophy. Auditory brainstem response (ABR) revealed moderate bilateral conductive hearing loss. Speech was limited to “baba,” which he said to everyone, and he followed only a few simple commands. At the age of 21 months, an ophthalmological examination revealed mild hypertelorism, bilateral lens opacities, and bilateral spoke‐wheel‐like opacities. Hyperopia (farsightedness) of the left eye was detected through cyclorefraction testing. Additionally, an unsteady gait and easy falling were noted in the neurological examination. Brain MRI indicated no abnormalities except for J‐shaped sella turcica. The skeletal X‐rays were suggestive of dysostosis multiplex. He started walking at the age of 20 months with support and had been following physiotherapy since the age of 4 years. Ultrasound confirmed hepatosplenomegaly.

Urine mucopolysaccharides (dermatan sulfate, heparan sulfate, keratan sulfate, and chondroitin‐6‐sulfate) were within normal range. The urine oligosaccharide profile was consistent with a biochemical diagnosis of AM. Alpha‐mannosidase activity in leukocytes was absent. *Genetic testing*: At the age of 2 years, ES identified a homozygous nonsense variant c.2368C> T (p.Gln790Ter) in *MAN2B1*. He had a normal chromosomal microarray. *Therapy and follow‐up*: ERT was initiated for the patient at 2 years old, followed by BMT performed abroad from a (10/10) HLA–matched unrelated donor at the age of 3 years with no major complications. ERT was terminated posttransplantation. GVHD prophylaxis included rituximab (Days 240–300 post‐BMT), cyclosporine A (Days −3 to 270 post‐BMT), mycophenolate mofetil, and abatacept. Standard *Pneumocystis jirovecii* pneumonia (PJP) prophylaxis and voriconazole were also given for a few months. He had several bacterial and viral infections after BMT. Low IgG was observed 1 year after BMT, requiring monthly doses of IVIG. Mild improvement in hearing impairment was noted by the family, and speech therapy was followed up.

### 3.4 Family 4: Siblings (Cases 4.1 and 4.2)

#### 3.4.1 Case 4.1

This is the case of a 27‐year‐old Emirati female. Her family history includes two other sisters with glycogen storage disease Type I. Hearing loss was noted at around 6 months of age, and the patient was eventually diagnosed with sensorineural hearing loss, with bilateral permanent T‐tubes. Adenoidectomy and tonsillectomy were performed. The parents reported recurrent ear and chest infections. Visual acuity was 20/100 for both eyes; she had blurred vision and photophobia. Her IQ score was very low (approximately 40–55), and she was managed with special education. She had the ability to walk, dress, and climb stairs independently but depended on her mother for bathing and eating. She had a short stature, scoliosis, and osteopenia, confirmed through DEXA scan. An echocardiogram showed trace mitral regurgitation with normal biventricular function and dimensions. She had hypothyroidism, managed with levothyroxine supplementation.

*Genetic testing*: At 13 years of age, mutation analysis revealed a homozygous, pathogenic nonsense mutation c.2368C> T (p.Gln790Ter) in *MAN2B1*; thus, confirming the diagnosis of AM. The family declined ERT.

#### 3.4.2 Case 4.2

A 23‐year‐old female was diagnosed with AM. Hearing loss was noted at 9 months of age and was linked to recurrent ear infections. Additionally, she also had recurrent chest infections. A limited speech of around 10 words was noted, but she could not formulate sentences. Sleep difficulties, behavioral changes, and aggression toward siblings prompted psychiatric consultation. She attended school and left at the age of 13. Visual acuity for both eyes was 20/100. Phacoemulsification of cataract with intraocular lens implant into the right eye was performed. Periodic orthopedic assessments revealed mild and nonprogressive scoliosis. The autoimmune thyroiditis was managed with l‐thyroxine. Trace mitral regurgitation was noted on an echocardiogram.

*Genetic testing*: The genetic variant found in her sibling (**Patient 4.1**) triggered further family screening, and mutation analysis at 10 years revealed a homozygous variant in *MAN2B1* c.2368C>T (p.Gln790Ter). Like her older sibling, ERT and BMT were not initiated.

### 3.5 Family 5

#### 3.5.1 Case 5.1

This is the case of a 9‐year‐old Emirati male with a family history that includes two healthy siblings, a maternal cousin with a low IQ and a paternal cousin with poor concentration. According to his medical records, he had talipes calcaneovalgus at birth. Additionally, he had coarse facial features, hypertelorism, prominent eyes, a depressed nasal bridge, a simple left ear helix, plagiocephaly, joint restrictions—bilateral shoulder external rotation restriction and bilateral elbow extension restriction—and hirsutism.

He was hospitalized multiple times because of recurrent otitis media and pneumonia; consequently, he had adenoidectomy, bilateral grommet insertion, and tonsillectomy at the ages of 1, 2, and 4 years, respectively. Moreover, he had bilateral hearing loss at 2 years of age. An audiometry test confirmed mixed and sensorineural hearing losses in the right and left ears, respectively. In addition, the patient had learning difficulties and speech delay at the age of 5 years, requiring speech therapy and special education. Social development appeared to be normal regarding play, reciprocal interaction including verbal and nonverbal communication, having friends, and a good range of emotional expression. He had minimal weakness of the left lower limb with weak stepping reflexes. Furthermore, a neurological test showed balance problems and poor fine motor skills. He had a delay in walking that was overcome with physiotherapy evident by the ability to run and walk at the age of 5 years. An echocardiogram showed a thickened anterior mitral leaflet with trivial mitral regurgitation.

*Genetic test*: The molecular testing workup included negative fragile X syndrome test and a chromosomal microarray result with CNV of unknown significance at the 19q13.12 region. At 5 years of age, ES revealed a homozygous, nonsense variant c.2119C> T (p.Gln707Ter) in *MAN2B1*, confirming AM. This case was reported by Saleh et al. as a part of a cohort of patients with neurogenetic disorders without complete clinical characterization [5]. Biochemical profile showed high levels of urine oligosaccharides and absent alpha‐mannosidase activity in leukocytes. The parents declined ERT.

### 3.6 Family 6: Siblings (Cases 6.1 and 6.2)

#### 3.6.1 Case 6.1

A 20‐year‐old Emirati male who was prematurely born at 8 months of pregnancy due to placental insufficiency. He presented at 6 months of age with otitis media and later developed psychomotor development delay, speech delay, and hearing impairment.

*Genetic testing*: Mutation analysis revealed homozygous, pathogenic nonsense mutation c.2119C> T (p.Gln707*) in *MAN2B1*.

*Treatment and follow‐up*: He underwent an allogeneic BMT at 3 years of age from an unrelated donor (mismatched unrelated donor 9/10); this was complicated by GVHD primarily affecting the face. Posttransplant evaluations showed full bone marrow engraftment.

He started his first grade at a mainstream school at 9 years of age with average performance. He demonstrates limited abstract thinking and has difficulty initiating and terminating conversations owing to poor language structure and lack of problem‐solving skills. However, he is reportedly interactive at school in the absence of constant supervision. The patient's health status at the last follow‐up was stable. He was cooperative, interactive, and content inside the clinic. Although he was shy, he maintained good eye contact with occasional gaze avoidance. The patient consistently responded to his name, talked clearly with only some ambiguities, and participated in tabletop activities. A multidisciplinary approach, including speech therapy, behavioral support, and academic accommodations, were recommended for comprehensive care.

Furthermore, an audiogram revealed mild to moderate hearing impairment. A brain MRI revealed generalized prominence of the right hemispheric sulci, cerebellar fissure, and bilateral optic nerve tortuosity. A spine MRI showed mild cervical C3 vertebral body height loss and minimal posterior disk bulge (C5/C6). His motor skills were normal, considering that he could independently perform daily activity with steady balance. However, he did not have any further follow‐up; therefore, no additional information can be retrieved.

#### 3.6.2 Case 6.2

A 14‐year‐old male Emirati male with an older sibling affected with AM (Section 3.6.1). He was diagnosed at birth because of a positive family history. He had recurrent episodes of pneumonia, upper respiratory tract infections, and was noted to have lower limb deformity in his first year in life.

*Genetic testing*: Targeted familial mutation analysis revealed a homozygous, pathogenic nonsense mutation c.2119C> T (p.Gln707*) in *MAN2B1*.

*Treatment and follow‐up*: At 1 year of age, he underwent an allogeneic hematopoietic stem cell transplant (HSCT) from an unrelated donor (5/6) in the United States. The cell dose was 0.48 × 10^8^ total cells/kg. He fared well, apart from Grade 1 GVHD in the skin and the development of bacteremia (*Mycobacterium chelonae*) with pulmonary involvement, requiring long‐term antibiotic use. He was conditioned with busulfan, cyclophosphamide, and antithymocyte globulin. He received GVHD prophylaxis with cyclosporine and mycophenolate mofetil.

Diagnosed initially with Hashimoto thyroiditis, requiring thyroxine supplementation, which was stopped at the age of 4 years. However, hyperthyroidism later developed, and Graves' disease was diagnosed at 7 years requiring thyroidectomy at 11 years and subsequent thyroxine supplementation. Physical examination revealed coarse features of a prominent forehead and downward‐slanting palpebral fissures. Additional skeletal abnormalities, including pectus carinatum, genu valgum, and scoliosis, were reported. The latest patient's pulmonary records indicated that the chest wall deformity (pectus carinatum) had been worsening with time.

At 14 years of age, the 6MWT in room air showed that he was ambulating 362.41 m with SpO_2_ ranging from 93% to 98% and HR ranging from 110 to 126 bpm. His performance was 52.8% of the age‐predicted norm as per the equation by Ulrich et al. (2013). Records at 15 years demonstrated that he was enrolled in school online and doing well. At 15 years, pulmonary clinic records revealed worsening chest defects and a recent diagnosis of pleuroparenchymal fibroelastosis (PPFE), managed with pirfenidone.

## 4 Discussion

This study documents the wide spectrum of clinical presentations associated with AM in the UAE, consistent with those reported worldwide [8, 13, 16]. Hearing loss and developmental delay are the predominant features of the disease observed in all patients. Hearing loss has a sensorineural component in some patients. Additionally, recurrent otitis media in some patients led to conductive hearing loss or a mixed type of conductive and sensorineural hearing loss. Speech is often delayed due to hearing loss and the underlying global developmental delay [12]. Hearing loss is usually nonprogressive in AM [8] and is considered the primary symptom in patients across various age groups [33]. Speech delay and intellectual disabilities (evident in low IQ test) are the primary psycho‐cognitive development delays in AM [16, 34, 35]. Psychiatric symptoms—such as aggression, depression, and psychosis—were evident in two patients. This aligns with several AM studies presenting psychiatric symptoms in adolescence [36].

Motor developmental delay (including fine and gross motor impairment or delay, and ataxia) is the second component in global developmental delay and is clearly reported in six patients here. Skeletal abnormalities (e.g., scoliosis and joint deformity) further impair mobility in these patients [4, 20]. 3MWT/6MWT and SARA (The Scale for the Assessment and Rating of Ataxia) are physical tests that are used to monitor patient motor function and are recommended every 6–12 months in pediatric patients [14]. Posttransplantation 6MWT of Section 3.6.2 indicated that the performance was 52% of the norm after 13 years of receiving therapy. Another feature of AM is macrocephaly [37, 38]; however, Patient 3.1 experienced microcephaly due to concurrent genetic diseases, structural heart defects, and renal anomalies (OMIM: 617478, *TMEM260*). Relatedly, coarse facies is one of the primary features of AM [33], observed in eight patients here.

Some patients may experience autoimmune disease due to AM, including thyroiditis, which was recorded in three patients. Moreover, hepatosplenomegaly is reported in some patients with AM [4, 19]. One out of the nine cases in our study had hepatosplenomegaly. Recurrent infections were reported in all patients, either in the form of respiratory or ear infections, which usually present in childhood and are considered one of the prominent symptoms of AM [33].

**FIGURE 3:**
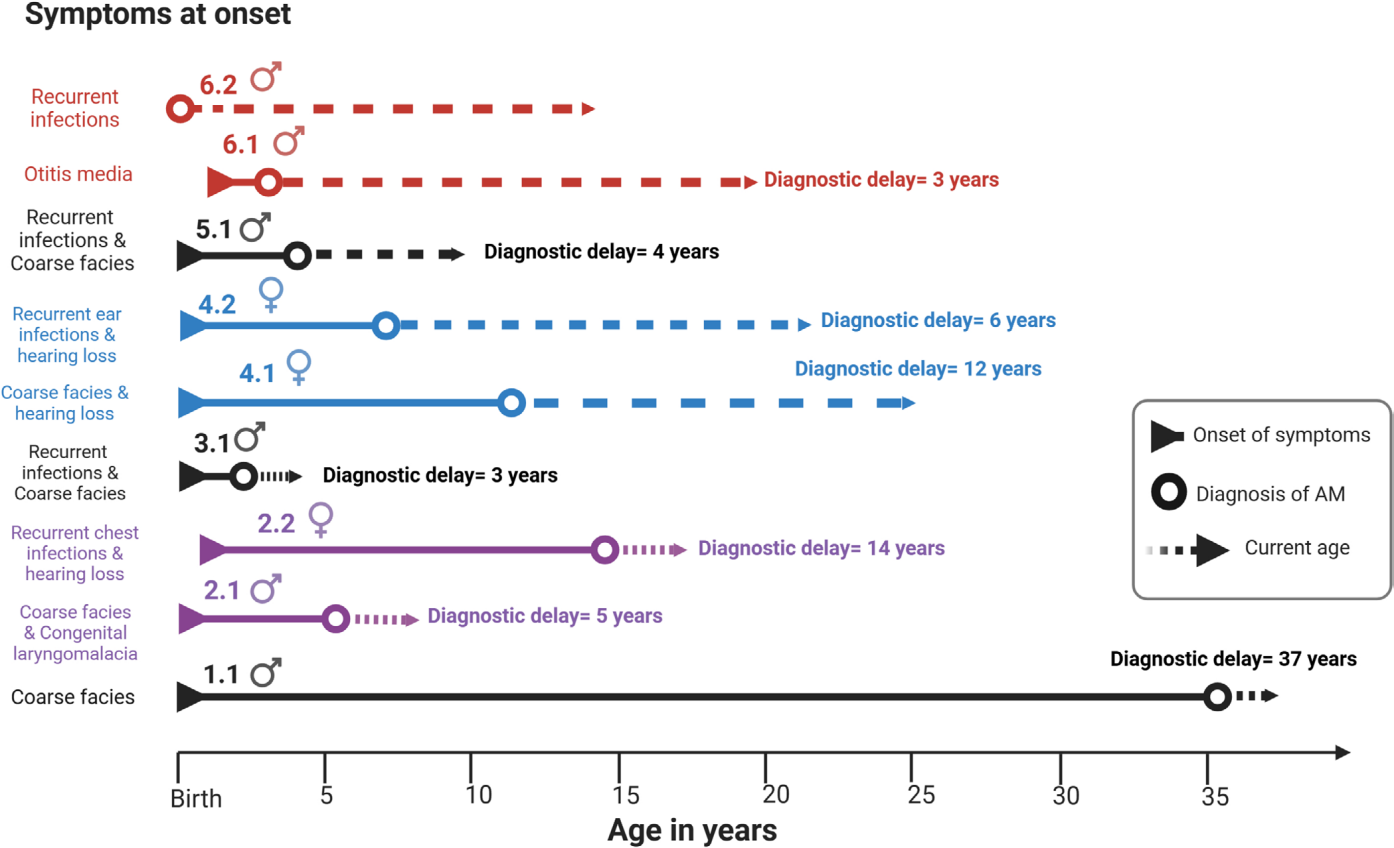
Diagnostic delay in alpha‐mannosidosis (AM). Each arrow represents one patient with the corresponding case number on top. The triangle at the beginning indicates the onset of AM symptoms. The open circle indicates the time of AM diagnosis. The remaining arrow travels to show the current age. Created in BioRender. https://BioRender.com/i33e312.

Developmental delay, hearing loss, coarse facies, and/ or recurrent infection are the main symptoms at presentation during the early years of life in this cohort (Figure 3). The onset and severity of symptoms vary widely in AM [16]. The cases in this cohort had an infantile onset of presentation, but the course of their disease was more consistent with the attenuated‐moderate form of the disease.

Diagnosis is usually delayed (Figure 3). The mean age of diagnosis is 14 years in adults (> 16 years) and 2 years in pediatric patients. Such a large difference in the mean age of diagnosis between older and younger patients is explained by improvements in genetic testing over the last decade. Advances in NGS and the increasing availability of genetic testing have enabled earlier diagnoses and access to therapy. A recent long‐term follow‐up study reported an average age of diagnosis of 6 years for AM, with an age range from 1–8 months to 12 years [8]. Moreover, recently proposed diagnostic algorithms can facilitate the early detection of this disease [33]. Another factor that shortens the diagnostic delay is a positive family history, and Sections 3.2.2, 3.4.2., and 3.6.2. are clear examples of this. Integrating genomic sequencing into newborn screening programs has the potential to significantly enhance the early identification of treatable rare diseases. The *MAN2B1* gene has been included in the disease list by the Genome‐to‐Treatment (GTRx) management guidance system [39, 40]. Furthermore, premarital screening and counseling ultimately play a major role in reducing the frequency of the disease [41]. Therefore, genetic mutation analysis of 570 genes, including *MAN2B1*, is included in the updated, mandatory premarital screening program for Emiratis planning to marry [42].

Consanguinity plays a major role in the probability of developing genetic disease, including AM [5, 6, 7]. Consanguinity is higher in the Middle East, including the UAE, compared to the global records [5, 6, 7]. The prevalence of AM, based on European data in the latest Orphanet report in 2024, is projected to be one in a million. Based only on this cohort, the birth prevalence of AM among Emiratis is estimated to be 1:100 000 live births for the period between 1980 and 2016, according to the previous method of calculating prevalence by Poorthuis et al. (1999), which has been used in other AM studies [7, 29, 30, 31]. Prevalence was 0.38 per 100 000 live births in Czech Republic (between 1985 and 1992) and 0.1 per 100 000 live births in Australia (between 1980 and 1996). All patients in this cohort are offspring of consanguineous marriages, and as expected, they have homozygous variants, indicating that the same variants were inherited from both parents. Notably, two variants (namely, NM_000528.3 c.2368C> T and NM_000528.3 c.2119C> T) appeared in two unrelated families according to our recordings, as noted in Table 1. These families may share common founder mutations as a product of high consanguinity [43, 44]. The same variants have been reported in other studies in the UAE [7, 45, 46]. NM_000528.3 c.1929‐2 A> G was also reported in a patient from Saudi Arabia [46].

ERT is one therapeutic option for treating non‐neurological manifestations of AM. ERT has a well‐tolerated safety profile, which alleviates several clinical features of AM, such as recurrent infections [22]. It could be also used as a bridging treatment to stabilize patients before undergoing HSCT. The national health insurance (Thiqa) covers both ERT and transplant treatments for Emirati. While all patients in this cohort were previously sent abroad for transplants, a transplant center has recently been established within the country. ERT therapy is expensive, and some families are unable to afford it without insurance support. Three patients in this cohort received ERT, which was reported to be well tolerated and effective in improving the immunological profile. To address neurological symptoms of the disease, three patients received transplantation. Section 3.6.2 received early umbilical cord blood (UCB) transplantation at the age of 1 year. He exhibited good intelligence and performed well in mainstream education at 15 years of age. Similarly, his affected sibling attended mainstream education but with difficulties. He had good motor skills, and his sociability improved after receiving a BMT at the age of 3 years. It can be concluded that BMT/UBCT was associated with better performance in motor and cognitive skills in **Patients 6.1** and **6.2**. **Patient 3.1** recently underwent a transplant, so there is insufficient data to draw any conclusions at this time. A comprehensive study is needed to assess the cost‐effectiveness of transplantation compared to ERT.

The posttransplant complications in our cohort included early GVHD and bacteremia in Section 3.6.2. Additionally, late‐onset PPFE was observed in that patient at the age of 17 years, that is, 14 years after transplantation [47, 48, 49, 50]. PPFE is one of the late‐onset noninfectious pulmonary complications that can manifest more than 3 months after transplantation [51]. Different antifibrotic agents have shown variable success in slowing the progression of the disease, including pirfenidone (which is prescribed for the patient); nevertheless, lung transplantation is considered the only life‐sustaining therapy [51, 52, 53]. PPFE can be worsened by restrictive skeletal defects [49, 54]. The patient had experienced progressive, restrictive skeletal defects, including pectus carinatum, since the age of 3 years. Furthermore, this patient received (5/6)‐HLA‐antigen–mismatched transplantation, which is considered a risk factor for chronic GVHD and hence PPFE [55, 56]. Relatedly, his sibling, who received 9/10‐mismatched BMT, had early‐onset GVHD affecting the face. Evidently, a 6/6 HLA antigen allelic match has less resolution, or a lesser antigen match, than a 9/10 or 10/10 allelic match.

## 5 Conclusions and Recommendations

Approximately 1 in 100 000 Emiratis suffer from AM, which is approximately five times higher than the global average. All patients in our study were born to consanguineous parents; therefore, all of them predictably had homozygous variants. The clinical presentation and symptom severity varied between patients; however, hearing loss, speech delay, and recurrent infections were consistently observed. Early intervention relies on early diagnosis, which is challenging for such rare diseases. Although this study offers valuable insights into AM in the UAE, it is limited by its retrospective nature and limited access to patient information beyond certain hospitals where ethical approval was obtained. Therefore, more comprehensive, prospective analyses of AM in the UAE, in addition to careful follow‐ups, could potentially identify age‐dependent symptoms and explore the therapeutic benefits of ERT or BMT/HSCT.

## Author Contributions

Fatma Al Jasmi conceived and managed the funding of this study, and designed the study. Tasneem Al‐Hammadi and Shaikha Al‐Ameri collected the data. Patient examinations were done by Aisha Al‐Shamsi, Noura Al‐Dhaheri, Amal Al Tenaiji, and Fatma Al Jasmi. Ali K. Saad analyzed the data and wrote the manuscript. All authors reviewed and approved the manuscript.

## Ethics Statement

This study was approved by the Abu Dhabi Health Research and Technology Committee, reference number DOH/CVDC/2023/834. Affected cases were identified by the metabolic teams at Tawam Hospital and Sheikh Khalifa Medical City, Abu Dhabi, UAE, for a retrospective review of patient electronic health records. Any clear patient identifiers were removed from this cohort.

## Conflicts of Interest

Amal Al Tenaiji and Fatma Al Jasmi have received consulting fees and Honoria from Biologix and Chiesi. The remaining authors declare no conflicts of interest.

## Data Availability

The authors have nothing to report.
